# Liver transplantation for alcoholic hepatitis: A systematic review with meta-analysis

**DOI:** 10.1371/journal.pone.0190823

**Published:** 2018-01-11

**Authors:** Astrid Marot, Margaux Dubois, Eric Trépo, Christophe Moreno, Pierre Deltenre

**Affiliations:** 1 Division of Gastroenterology and Hepatology, Centre Hospitalier Universitaire Vaudois, University of Lausanne, Lausanne, Switzerland; 2 Department of Gastroenterology, Hepatopancreatology, and Digestive Oncology, CUB Hôpital Erasme, Université Libre de Bruxelles, Brussels, Belgium; 3 Laboratory of Experimental Gastroenterology, Université Libre de Bruxelles, Brussels, Belgium; Universidad de Navarra, SPAIN

## Abstract

**Background:**

The rate of alcohol relapse among patients who underwent liver transplantation for alcoholic hepatitis (AH) is not precisely known.

**Aim:**

Synthesize the available evidence on liver transplantation for AH to assess alcohol relapse and 6-month survival.

**Methods:**

Meta-analysis of trials evaluating liver transplantation for AH, either clinically severe or diagnosed on the explant.

**Results:**

Eleven studies were included. The pooled estimate rate for alcohol relapse was 0.22 (95% CI = 0.12–0.36) in overall analysis with high heterogeneity between studies (*I*^*2*^ = 76%), 0.20 (95% CI = 0.07–0.43) in the subgroup analysis including patients with clinically severe AH (*I*^*2*^ = 84%), 0.14 (95% CI = 0.08–0.23) among patients with clinically severe AH in sensitivity analysis excluding the discrepant studies that did not use stringent selection criteria for liver transplantation (*I*^*2*^ = 0%), and 0.15 (95% CI = 0.07–0.27) for recurrent harmful alcohol consumption among patients with clinically severe AH (*I*^*2*^ = 3%). The risk of alcohol relapse was not different between AH transplanted patients and patients with alcoholic cirrhosis who underwent elective liver transplantation in sensitivity analysis excluding the discrepant studies (OR = 1.68, 95%CI = 0.79–3.58, p = 0.2, *I*^*2*^ = 16%). The pooled estimate rate for 6-month survival was 0.85 (95% CI = 0.77–0.91, *I*^*2*^ = 49%), and 0.80 among patients transplanted for clinically severe AH (95% CI = 0.69–0.88, *I*^*2*^ = 30%). AH transplanted patients had similar 6-month survival to patients with alcoholic cirrhosis who underwent elective liver transplantation (OR = 2.00, 95% CI = 0.95–4.23, p = 0.07, *I*^*2*^ = 0%).

**Conclusion:**

Using stringent selection criteria, 14% of patients with clinically severe AH have alcohol relapse after liver transplantation. The percentage of alcohol relapse of AH transplanted patients is similar than that of patients who underwent elective liver transplantation.

## Introduction

The treatment of severe forms of alcoholic hepatitis (AH) remains a challenge, especially for non-responders to corticosteroids who only have a 25% survival probability at 6 months [1]. In the absence of another therapeutic option, liver transplantation has been proposed in this setting. In a recent study, the 6-month survival of patients with a first episode of severe AH not responding to medical therapy who underwent early liver transplantation was significantly better than that for non-responders who were not transplanted [2]. This study challenged the rule that a 6-month period of abstinence should be required before considering liver transplantation for patients with alcoholic liver disease.

The reasons why a 6-month abstinence period is usually applied before considering a patient with alcoholic liver disease for liver transplantation are manifold. They include hope that liver function will improve after alcohol withdrawal, fear of recurrent alcohol consumption after liver transplantation, organ shortages, and fear that considering liver transplantation in patients with a self-inflicted disease could raise a problem of equity in liver graft allocation and in both public opinion and healthcare providers who participate in candidate selection [3, 4]. However, a recent survey indicated that early liver transplantation for carefully selected patients with acute AH was not as controversial with the public as previously thought [5]. In addition, only a few patients with severe AH returned to alcohol consumption after liver transplantation in recent studies that applied strict selection criteria for choosing candidates [2, 6, 7]. Finally, previous studies among patients with alcoholic cirrhosis have indicated that the 6-month rule of abstinence poorly identifies patients with recurrent alcohol consumption after liver transplantation [8, 9]. In the setting of liver transplantation for patients with AH, in addition to the studies performed in patients with severe AH, a couple of studies have also identified AH on explants from patients with alcoholic liver disease, suggesting that these patients probably did not fulfill the 6-month abstinence rule before liver transplantation [10, 11]. These studies provide additional data on alcohol relapse following liver transplantation for AH.

Despite the large amount of data on liver transplantation for AH that already exists, the rate of recurrent alcohol consumption after liver transplantation among patients with AH is still not precisely known. Meta-analysis is a quantitative technique that enables pooling data from trials in order to decrease random error. It also allows for assessment of the magnitude of impact of a particular factor. In this study, we performed a meta-analysis of trials evaluating liver transplantation among patients with AH. Our main objective was to assess the rate of alcohol relapse among AH transplanted patients and to compare it to alcohol relapse rates among patients with alcoholic cirrhosis who underwent elective liver transplantation. Our secondary objectives were to assess the rates of harmful alcohol relapse and 6-month survival among AH transplanted patients, and to compare 6-month survival rates among AH transplanted patients to those of non-responders who were not transplanted or to patients with alcoholic cirrhosis who underwent elective liver transplantation.

## Materials and methods

### Literature search

Medline (PubMed), Embase, Cochrane library, and manual searches were combined and last performed on June 10^th^, 2017. Key search terms were “alcoholic hepatitis”, “abstinence”, “alcohol relapse”, and “liver transplantation”. Terms were combined within each database. General reviews and references from published trials were also used. The exact search term combinations can be found in the S1 Appendix. Duplicate were excluded. No language restriction was applied. Two observers (A.M. and M.D.) also screened all abstracts presented between 2014 and 2017 at the Liver Meeting of the American Association for the Study of Liver Diseases (AASLD) and the International Liver Congress of the European Association for the Study of the Liver (EASL).

### Criteria for inclusion and exclusion of studies

All observational studies were included. In order to reduce risk of bias, strict inclusion and exclusion criteria were defined prior to the literature search. To be considered, a study had to: a) include patients with AH, either patients with acute jaundice and severe AH or patients with alcoholic cirrhosis who underwent a liver transplantation and in whom the diagnosis of AH was made on the explant; b) provide data relative to alcohol consumption or survival after liver transplantation. When several publications were found covering the same study population, only the most recent was taken into account.

### Endpoints

Endpoints were defined prior to the beginning of the meta-analysis. The main endpoint was alcohol relapse defined as any alcohol consumption after liver transplantation. The secondary endpoints were harmful drinking defined as binge, frequent, regular or daily drinking, and 6-month survival.

### Data extraction

Data extraction was performed independently by two investigators (A.M. and M.D.) using standardized data collection forms. Discrepancies in data interpretation were resolved by discussion, re-review of the studies and consultation with one other author (P.D.) when necessary.

### Quality score

The methodological quality of studies was assessed using the Newcastle Ottawa Scale for cohort studies [12].

### Statistical analysis

We used a random effects model to obtain a summary estimate of primary outcomes among patients with AH. The random-effects model was chosen because it takes into account the possibility of heterogeneity between studies [13]. Data on all patients were extracted to allow intention-to-treat analyses. We calculated event rates among patients with AH, a measure of how often a particular statistical event occurs within a group in an experiment, with 95% confidence intervals (CI), as already done elsewhere [14–16]. Differences between groups are expressed as odds ratios (ORs) with 95% CIs. A p-value <0.05 was considered statistically significant.

As a first step, an overall meta-analysis was performed. This analysis included transplanted patients with clinically severe AH and transplanted patients in whom the diagnosis of AH was made on the explant. In a second step, subgroup analyses in patients with and without clinically severe AH were performed. The overall analysis also included studies comparing AH transplanted patients to non-responders to medical therapy who were not transplanted, or to patients with alcoholic cirrhosis but without AH who underwent elective liver transplantation. In a third step, subgroup analyses comparing AH transplanted patients to non-responders to medical therapy who were not transplanted or to patients with alcoholic cirrhosis but without AH who underwent elective liver transplantation were performed.

Heterogeneity was assessed by Cochran's Q test [17] and the *I*^*2*^ statistic. More specifically, the *I*^*2*^ statistic was used to estimate inconsistency in the meta-analysis, representing the percentage of the between-study variability due to heterogeneity rather than chance [18]. A significant Cochran’s Q-statistic (below 0.10) was chosen as a threshold for significant heterogeneity across studies. The following cut-offs were used to quantify heterogeneity with the *I*^*2*^ statistic: 0–25%, low; 25–50%, moderate; >50%, high heterogeneity [18]. In cases of moderate or high heterogeneity, the methodological section of each study was re-reviewed to determine whether any discrepancy could be identified, and sensitivity analyses excluding the discrepant study were performed. To assess the extent of publication bias, the Egger test, the Begg and Mazumdar test and the *Trim and Fill* adjustment were used [17, 19]. A p-value <0.05 was considered statistically significant. All statistical analyses were performed using Comprehensive Meta-analysis (Biostat, Englewood, NJ).

## Results

### Study population

**Fig 1:**
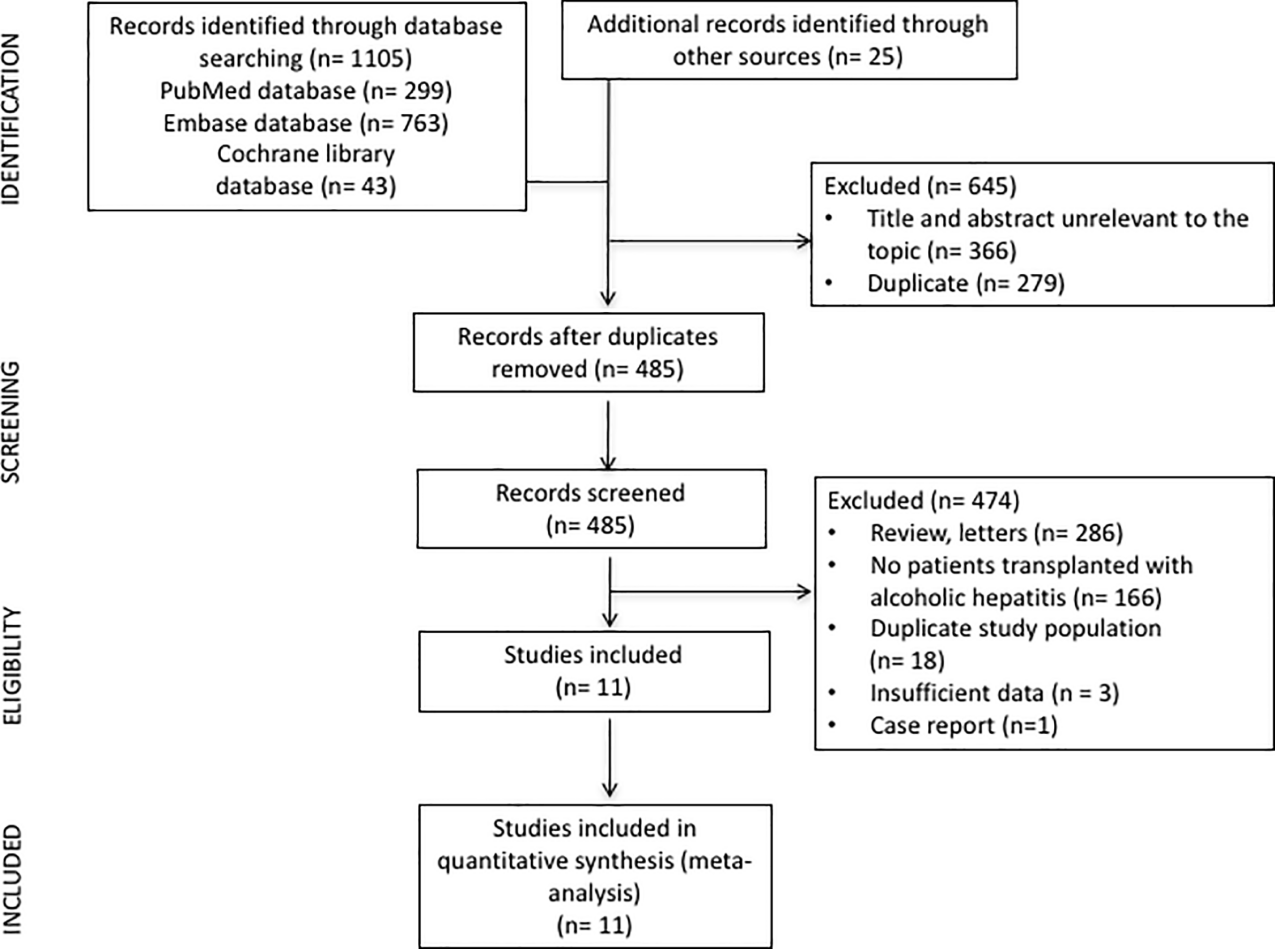
Flow chart of the selection of studies for inclusion in the meta-analysis.

Fig 1 summarizes the flow chart of the selection of studies for inclusion in the meta-analysis. We screened 1130 references; 485 were selected for full-text retrieval. Of these, 11 were included in the analysis [2, 7, 10, 11, 20–26]. One study [27] which was a national registry that included patients from 2 other studies [7, 21] was excluded to avoid duplication of data.

Table 1 summarizes the main characteristics of the studies included in the meta-analysis. A total of 325 patients with AH were included. There were 240 patients with clinically severe AH and 85 patients in whom the diagnosis of AH was made on the explant.

**Table 1:** Characteristics of the 11 included studies.

References	Study design	N of patients with AH	Type of AH	Age (years)	Male (%)	Histologically proven AH	Duration of abstinence before liver transplantation	Criteria for selecting patients for liver transplantation					MELD score	Maddrey DF
								First episode of AH	Good psychosocial support and/or favorable psychological profile	Non-response to medical therapy	Sobriety contract ^#^	N of criteria		
Hanouneh 2014 (20)	Retrospective	29	Severe	NA	NA	NA	NA	NA	Yes	Yes	Yes	3/4	35	110
Im 2016 (21)	Retrospective	16	Severe	41 * °	56% °	Yes	33 days before listing for liver transplantation ** °	Yes in 89% of patients °	Yes	Yes	Yes	4/4	39 *	92 * °
Immordino 2009 (10)	Retrospective	8	Diagnosed on the liver explant	53 °°	86% °°	Yes	NA	‒					14 °°	NA
Lee 2017 (7)	Retrospective	17	Severe	50 **	76%	Yes	40 days	Yes	Yes	Yes	Yes	4/4	37 *	67 * °°°
Mathurin 2011 (2)	Prospective	26	Severe	47 *	58%	Yes for most of the patients	<3 months	Yes	Yes	Yes	Yes	4/4	34 *	76 * °°°°
Shakil 1997 (22)	Retrospective	9 ^**##**^	Diagnosed on the liver explants	41 **	89%	Yes	≤1 month for 6 patients	‒					NA	47 **
Siddachari 2014 (23)	Retrospective	33	Severe	46 **	NA	NA	NA	NA	Yes	Yes	NA	2/4	30 **	91 **
Singal 2012 (24)	Retrospective	55	Severe	52 **	76%	No for most of the patients	<3 months	Well-selected group of AH patients					26 **	NA
Tomé 2002 (11)	Retrospective	36	Diagnosed on the liver explant	51 *	92%	Yes	15 months *	‒					NA	72% <32 28% ≥32
Van Thiel 1995 (25)	Retrospective	64	Severe	47 **	73%	Yes	All patients were active consumers	NA	No	NA	No	0/4	NA	NA
Wells 2007 (26)	Retrospective	32	Diagnosed on the explant	50 **	69%	Yes	9.7 months **	‒					21 **	38**

Abbreviations: NA, not available; AH, alcoholic hepatitis

* Expressed as median

** Expressed as mean

° Data extracted from ref. [6]

°° Data extracted from the whole study population transplanted

°°° Missing values in 2 patients

°°°° Data at the first day of medical therapy

# Sobriety contract means oral or written contract agreeing to stay abstinent of alcohol after liver transplantation

## 17 patients had typical histological features AH on the liver explants but only 9 patients fulfilled the criterion of severe AH with DF>32 and were analyzed in this study

### Study quality

S1 Table details the quality of the studies included.

### Methodological assessment of studies

With the exception of the Mathurin’s study [2], all studies were retrospective (Table 1). The methodological analysis of each study identified discrepancies in 2 of them [22, 25]. In these studies, either active consumers or patients abstinent only for a very short period of time were intentionally transplanted without using stringent criteria for selecting candidates for liver transplantation as has been done in the most recent studies (Table 1). These criteria included patients’ motivation to stay abstinent of alcohol following liver transplantation, a good psychosocial support and/or a favorable patient psychological profile. In cases of moderate or high heterogeneity in subgroup analyses, sensitivity analyses excluding the Shakil [22] and the Van Thiel [25] studies were performed.

### Outcomes

#### Risk of alcohol relapse after liver transplantation

**Fig 2:**
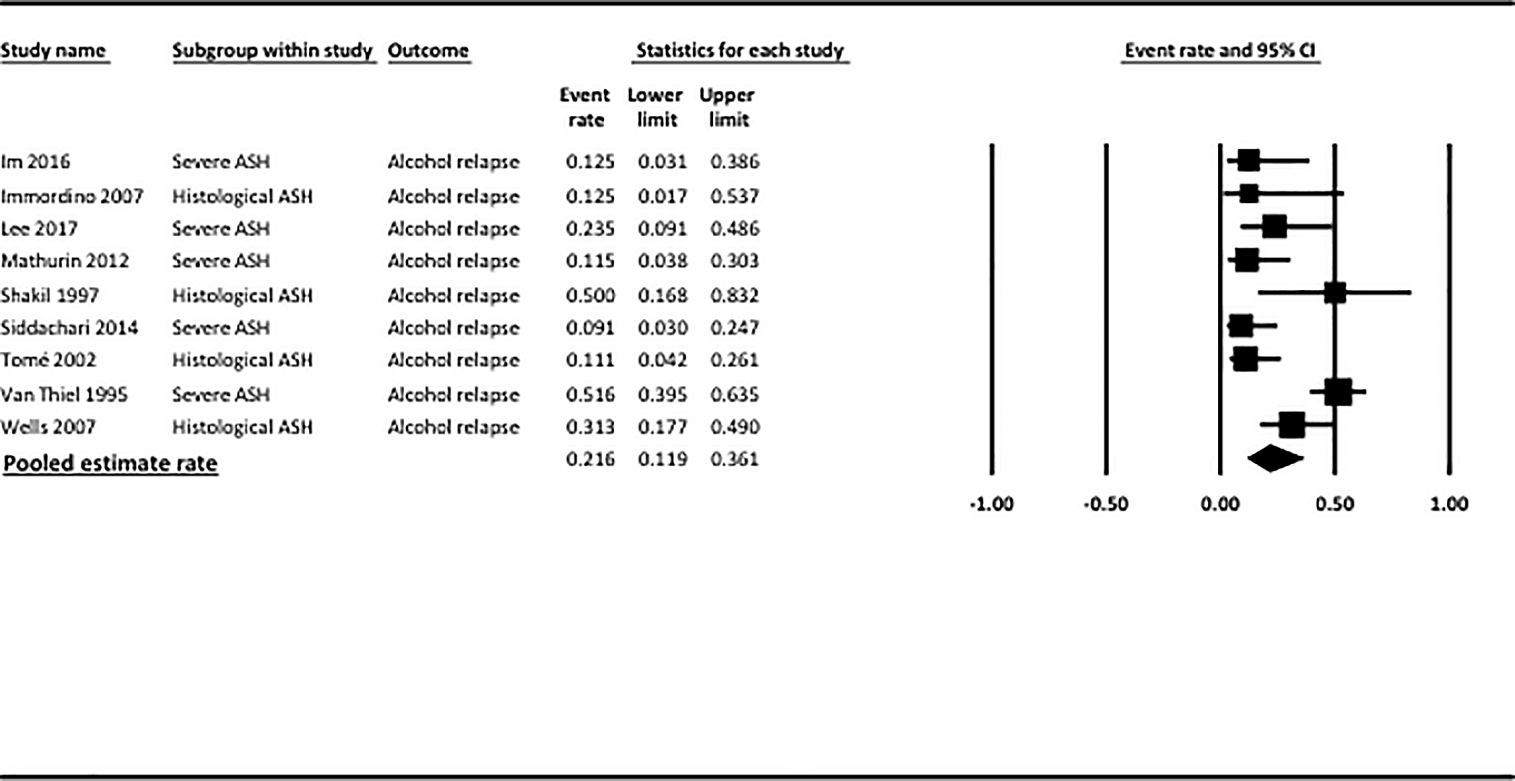
Pooled estimate rate of alcohol relapse after liver transplantation among AH transplanted patients. CI, confidence interval.

The pooled estimate rate for alcohol relapse was 0.22 (95% CI = 0.12–0.36, Fig 2 and Table 2). There was high heterogeneity between studies (p<0.001, *I*^*2*^ = 76%). Publication bias was detected by the Egger test (p = 0.02) but not by the Begg and Mazumdar test (p = 0.7). Funnel plot are shown in S1 Fig, including results using the *Trim and Fill* adjustment.

**Table 2:** Alcohol relapse and 6-month survival after liver transplantation among the 11 included studies.

References	Groups of patients	N	Alcohol relapse (n)	Harmful alcohol relapse (n)	6-month survival (%)
Hanouneh 2014 (20)	AH transplanted patients	29	NA^£^	NA	87%
Im 2016 (21)	AH transplanted patients	16	2	2^££^	88%
	Non-responders to corticoids who were not transplanted	152	‒	‒	19%
Immordino 2009 (10)	AH transplanted patients	8	1	NA	NA
	Patients with alcoholic cirrhosis who were transplanted	102	12	NA	NA
Lee 2017 (7)	AH transplanted patients	17	4	4^£££^	100%
	Patients with alcoholic cirrhosis who were transplanted	26	7°	3^£££^	88%
Mathurin 2011 (2)	AH transplanted patients	26	3	2^££££^	77%
	Non-responders to corticoids who were not transplanted	26	‒	‒	23%
Shakil 1997 (22)	AH transplanted patients	9	3°°	NA	89%
Siddachari 2014 (23)	AH transplanted patients	33	3	NA	70%
	Patients with alcoholic cirrhosis who were transplanted	127	11	NA	NA
Singal 2012 (24)	AH transplanted patients	55	NA	NA	95%°°°
	Patients with alcoholic cirrhosis who were transplanted	165	NA	NA	90%°°°
Tomé 2002 (11)	AH transplanted patients	36	4	NA°°°°	79%°°°
	Patients with alcoholic cirrhosis who were transplanted	32	3	NA°°°°	72%°°°
Van Thiel 1995 (25)	AH transplanted patients	64	33	NA	NA
	Patients with alcoholic cirrhosis who were transplanted	145	22	NA	NA
Wells 2007 (26)	AH transplanted patients	32	10	8^£££££^	96%°°°
	Patients with alcoholic cirrhosis who were transplanted	116	14	12^£££££^	86%°°°

Abbreviations: NA, not available; AH, alcoholic hepatitis

° Data among 24 patients (2 patients who died during the immediate postoperative period were excluded from this analysis)

°° Data on alcohol relapse available in only 6 patients

°°° Data extrapolated from survival curves

°°°° In ref. [11], data on harmful alcohol relapse are pooled for AH patients and patients with alcoholic cirrhosis

£ Discordant results in the abstract did not allow for extraction of accurate data

££ Alcohol relapse was defined as alcohol consumption of 4 or more drinks in a day or at least one drink for 4 or more days in succession after liver transplantation (harmful drinking)

£££ Harmful alcohol relapse was defined as alcohol relapse with binge drinking or frequent patterns defined as any alcohol consumption on 4 days in a week

££££ Harmful alcohol relapse was defined as daily alcohol consumption

£££££ Harmful alcohol relapse was defined as ≥6 alcoholic drinks in one day

Results of subgroup analyses including only patients with clinically severe AH are reported as Supporting Information (S2 Table). The pooled estimate rate for alcohol relapse was 0.20 (95% CI = 0.07–0.43). There was high heterogeneity between studies (p<0.001, *I*^*2*^ = 84%). In sensitivity analysis excluding studies that did not use stringent criteria for selecting candidates for liver transplantation, the pooled estimate rate for alcohol relapse was 0.14 (95% CI = 0.08–0.23), with no heterogeneity between studies (p = 0.6, *I*^*2*^ = 0%). The rate for harmful alcohol relapse was 0.15 (95% CI = 0.07–0.27), with negligible heterogeneity between studies (p = 0.4, *I*^*2*^ = 3%).

Results of subgroup analyses including only patients in whom the diagnosis of AH was made on the explant are reported as Supporting Information (S2 Table). The pooled estimate rate for alcohol relapse was 0.23 (95% CI = 0.11–0.43). There was high heterogeneity between studies (p = 0.09, *I*^*2*^ = 54%). In sensitivity analysis excluding studies that did not use stringent criteria for selecting candidates for liver transplantation, the pooled estimate rate for alcohol relapse was 0.19 (95% CI = 0.08–0.38), with high heterogeneity between studies (p = 0.1, *I*^*2*^ = 54%). The rate for harmful alcohol relapse was not assessed in this subgroup analysis due to the limited number of studies with available data.

**Fig 3:**
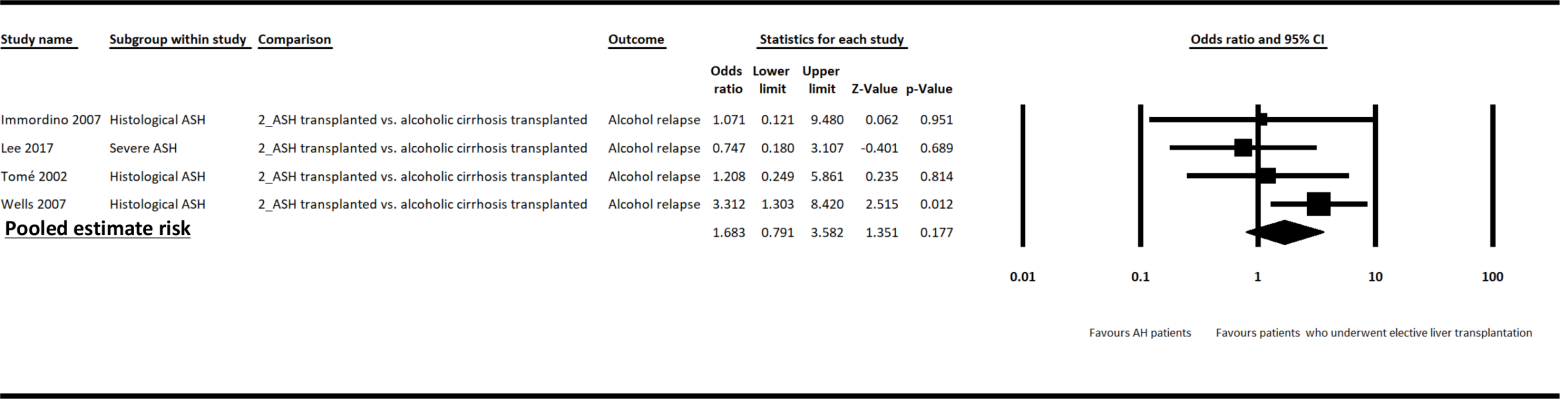
Risk of alcohol relapse after liver transplantation between AH transplanted patients and patients with alcoholic cirrhosis who underwent elective liver transplantation in sensitivity analysis excluding studies that did not use stringent criteria for selecting candidates for liver transplantation. CI, confidence interval; OR, odds ratio.

AH transplanted patients had a non-significantly different risk of alcohol relapse compared to patients with alcoholic cirrhosis who underwent elective liver transplantation (OR = 2.28, 95% CI = 0.98–5.29, p = 0.055), with high heterogeneity between studies (p = 0.05, *I*^*2*^ = 59%). In the sensitivity analysis excluding studies that did not use stringent criteria for selecting candidates for liver transplantation, AH transplanted patients had a similar risk of alcohol relapse compared to patients with alcoholic cirrhosis who underwent elective liver transplantation (OR = 1.68, 95% CI = 0.79–3.58, p = 0.2, Fig 3), with low heterogeneity between studies (p = 0.3, *I*^*2*^ = 16%). Subgroup analysis including only patients with severe AH was not done due to the limited number of studies with available data. In the subgroup analysis including only patients in whom the diagnosis of AH was made on the explants, the risk of alcohol relapse was higher among AH transplanted patients than in those who underwent elective liver transplantation (OR = 2.3, 95% CI = 1.08–4.89, p = 0.03), with no heterogeneity among studies (p = 0.4, *I*^*2*^ = 0%).

#### 6-month survival

**Fig 4:**
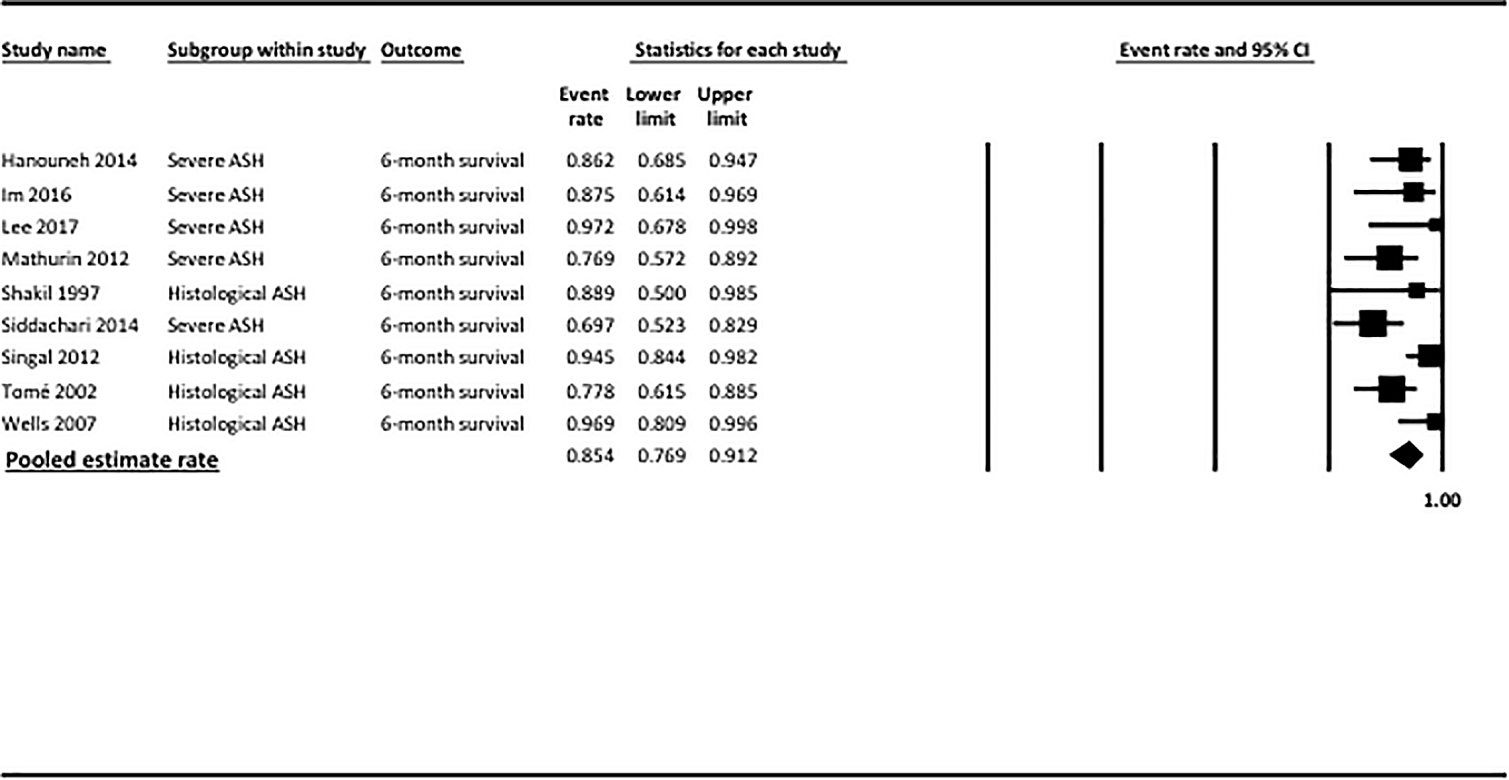
Pooled estimate rate for survival at 6 months among AH transplanted patients. CI, confidence interval.

The pooled estimate rate for 6-month survival was 0.85 (95% CI = 0.77–0.91, Fig 4 and Table 2). There was high heterogeneity between studies (p = 0.05, *I*^*2*^ = 49%). Publication bias was detected by the Egger test (p = 0.01) and by the Begg and Mazumdar test (p = 0.06). Funnel plot are shown in S2 Fig, including results using the *Trim and Fill* adjustment.

Results of subgroup analyses including only patients with clinically severe AH are reported as Supporting Information (S2 Table). The pooled estimate rate for 6-month survival was 0.80 (95% CI = 0.69–0.88), with moderate heterogeneity between studies (p = 0.2, *I*^*2*^ = 30%).

Results of subgroup analyses including only patients in whom the diagnosis of AH was made on the explant are reported as Supporting Information (S2 Table). The pooled estimate rate for 6-month survival was 0.90 (95% CI = 0.76–0.97), with high heterogeneity between studies (p = 0.06, *I*^*2*^ = 60%). In sensitivity analysis excluding studies that did not use stringent criteria for selecting candidates for liver transplantation, the pooled estimate rate for 6-month survival was 0.91 (95% CI = 0.72–0.98), with high heterogeneity between studies (p = 0.02, *I*^*2*^ = 73%).

AH transplanted patients had better 6-month survival than did non-responders to corticoids who were not transplanted (OR = 16.69, 95% CI = 6.21–44.81, p<0.001), with no heterogeneity between studies (p = 0.3, *I*^*2*^ = 0%).

AH transplanted patients had similar 6-month survival compared to patients with alcoholic cirrhosis who underwent elective liver transplantation (OR = 2.00, 95% CI = 0.95–4.23, p = 0.07), with no heterogeneity between studies (p = 0.7, *I*^*2*^ = 0%). Subgroup analyses including only patients with severe AH were not done due to the limited number of studies with available data. In the subgroup analysis including only patients in whom the diagnosis of AH was made on the explants, AH patients had similar 6-month survival compared to patients who underwent elective liver transplantation (OR = 1.88, 95% CI = 0.87–4.07, p = 0.1), with no heterogeneity among studies (p = 0.6, *I*^*2*^ = 0%).

## Discussion

The increasing recognition of liver transplantation as a rescue therapy for patients with severe AH not responding to medical therapy has challenged the 6-month rule of abstinence. This rule, which poorly identifies patients with alcoholic liver disease who will have recurrent alcohol use after liver transplantation [8, 9], is not applicable to patients with severe AH who have no other therapeutic option and who have a high risk of death within 6 months after the onset of the disease. Since the landmark study of Mathurin et al. in 2011 [2], several teams have reported their first experience with early liver transplantation among patients with severe AH. In addition, a couple of studies have reported on the outcomes of patients transplanted for alcoholic cirrhosis in which AH was discovered on the explants. Despite using a different selection process than the one used for patients with clinically severe AH, these studies provide additional data on the behavior of AH patients following liver transplantation. As a result, there are quite a lot of data currently available on the risk of alcohol relapse among AH transplanted patients. Thus, a meta-analysis is required to synthesize available data.

This meta-analysis found that only one-fifth of AH patients had alcohol relapse after liver transplantation. The percentage of AH transplanted patients with alcohol relapse was not significantly different than that of patients with alcoholic cirrhosis who underwent elective liver transplantation with the exception of patients in whom the diagnosis of AH was made on the explant. In these cases, proper selection of candidates for liver transplantation was less likely than in patients not responding to corticosteroids and who, in the end, were transplanted. Of note, after the exclusion of a discrepant study that did not use stringent criteria for selecting candidates for liver transplantation, only 14% of patients with clinically severe AH had alcohol relapse after liver transplantation. However, in cases of recurrence, most patients had harmful drinking patterns as has already been shown for patients transplanted for alcoholic cirrhosis after a 6-month sobriety period [28]. Overall, these results indicate that the selection of AH patients for liver transplantation should be based on stringent criteria, as has been done in the most recent studies [2, 6]. Currently, the use of such strict selection process seems to be a key factor that allows for selection of patients with a low risk of alcohol relapse after liver transplantation.

This meta-analysis confirmed that liver transplantation considerably improved the prognosis of patients with severe AH who were not responding to medical therapy. The pooled estimate rate for survival was 85% at 6 months among all AH transplanted patients and 80% among patients with clinically severe AH. This was much better than the rate for non-transplanted non-responder patients. In addition, AH transplanted patients had similar 6-month survival compared to patients with alcoholic cirrhosis who underwent elective liver transplantation. However, data on long-term prognosis were not available. This could be a matter of concern among AH patients with recurrent alcohol consumption as alcohol relapse is likely to impact long-term prognosis. Nevertheless, as liver transplantation can only be proposed to a limited number of patients, alternative pharmacological therapies should be developed for patients who are not responding to medical therapy to reduce the number of potential candidates for liver transplantation.

We acknowledge that this meta-analysis has several limitations. One set of limitations is related to the fact that AH was not confirmed with a liver biopsy in all patients with clinically severe AH, to the retrospective design of most studies, and to the limited number of patients. However, it was expected that most studies dealing with non-responder transplanted AH patients were retrospective and included few patients. In addition, individual participant data and data related to the effect of alcohol relapse according to the pattern of drinking would have been of interest but, unfortunately, they were not available. Another limitation is the existence of publication bias detected regarding the risk of alcohol relapse and 6-month survival, despite our efforts to limit this risk as much as possible using a number of methods. However, the impact of this bias was probably modest. Another issue lies with the criteria used for selecting patients for liver transplantation that were not uniform between studies. A classical limitation of meta-analyses is also related to the presence of heterogeneity that may prevent making robust conclusions and recommendations. This suggests that a substantial proportion of the difference in the effect between studies cannot only be explained by random sampling but by true differences between studies. In this meta-analysis, high heterogeneity was found for several analyses on the risk of alcohol relapse and survival. However, heterogeneity was always reduced and even disappeared in subgroup analyses or when discrepant studies that did not use stringent criteria for selecting candidates for liver transplantation were excluded. Therefore, the results of these analyses seem robust for assessment of the rate of alcohol relapse among AH transplanted patients even if they are based on a limited number of studies. Nevertheless, prospective studies using stringent selection criteria are still needed. In line with this, the first results of an ongoing prospective observation trial focusing on alcohol relapse after liver transplantation (NCT01756794) expected at the beginning of 2018 will be of interest. This study may contribute to the identification of factors predicting alcohol relapse after liver transplantation in daily practice.

In summary, 14% of carefully selected patients with clinically severe AH not responding to medical therapy have alcohol relapse after liver transplantation. When rigorous criteria for selecting candidates for liver transplantation are applied, the percentage of alcohol relapse of AH transplanted patients is not different than that of patients with alcoholic cirrhosis who underwent elective liver transplantation. Stringent selection criteria should be defined and applied when considering liver transplantation in patients with AH.

## Supporting information

S1 Fig Funnel plot of studies evaluating alcohol relapse. As the shape of the funnel plot for the studies was not symmetrical, the Duval and Tweedie’s *Trim and Fill* adjustment was used to estimate the extent of the impact of the bias and what the effect size would have been in the absence of bias. It re-computes the effect size at each iteration until the funnel plot is symmetric about the new effect size. The observed studies are shown as open white circles and the observed point estimate in log units is shown as an open white diamond, while the imputed studies are shown in red open circles and the imputed point estimate in log units is shown as a red diamond. Overall, the impact of bias was probably modest.
(TIFF)

S2 Fig Funnel plot of studies evaluating 6-month survival. As the shape of the funnel plot for the studies was not symmetrical, the Duval and Tweedie’s *Trim and Fill* adjustment was used to estimate the extent of the impact of the bias and what the effect size would have been in the absence of bias. It re-computes the effect size at each iteration until the funnel plot is symmetric about the new effect size. The observed studies are shown as open white circles and the observed point estimate in log units is shown as an open white diamond, while the imputed studies are shown in red open circles and the imputed point estimate in log units is shown as a red diamond. Overall, the impact of bias was probably modest.
(TIFF)

S1 Table Quality assessment of the 11 included studies using the Newcastle Ottawa Scale. NA, not applicable.
(DOCX)

S2 Table Results of subgroup analyses including only patients with clinically severe AH or in whom the diagnosis of AH was made on the explants. AH, alcoholic hepatitis; CI, confidence interval.
(DOCX)

S1 Appendix Search strategy used for the identification of eligible studies for the meta analysis. (DOCX)

## Abbreviations

AASLD: American Association for the Study of Liver Diseases
AH: alcoholic hepatitis
CI: confidence interval
EASL: European Association for the Study of the Liver
Maddrey’s DF: Maddrey’s discriminant function
NA: not available
OR: odds ratio
