# Supplementary figures and images for: Liver transplantation for alcoholic hepatitis: A systematic review with meta-analysis
Source: PLoS One. 2018 Jan 11;13(1):e0190823. doi: 10.1371/journal.pone.0190823 (PMC5764315; doi:10.1371/journal.pone.0190823)

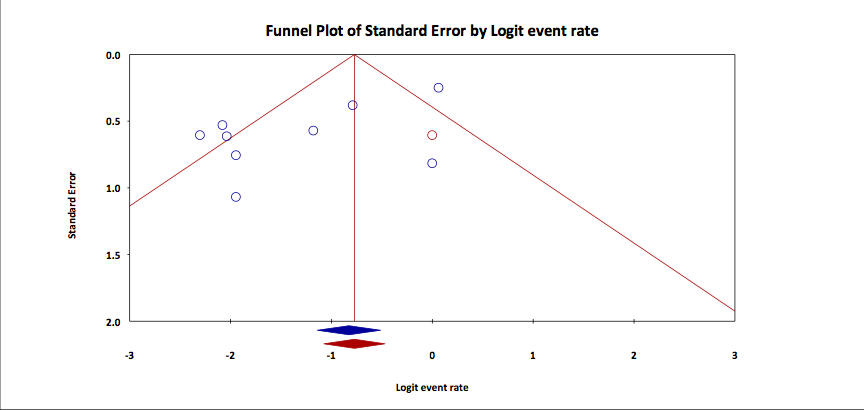

Supplement: S1 Fig — As the shape of the funnel plot for the studies was not symmetrical, the Duval and Tweedie’s Trim and Fill adjustment was used to estimate the extent of the impact of the bias and what the effect size would have been in the absence of bias. It re-computes the effect size at each iteration until the funnel plot is symmetric about the new effect size. The observed studies are shown as open white circles and the observed point estimate in log units is shown as an open white diamond, while the imputed studies are shown in red open circles and the imputed point estimate in log units is shown as a red diamond. Overall, the impact of bias was probably modest. (TIFF) [file pone.0190823.s001.tiff]

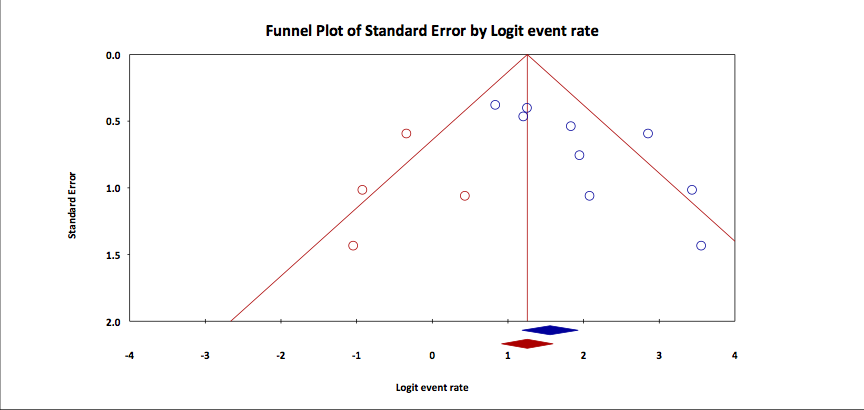

Supplement: S2 Fig — As the shape of the funnel plot for the studies was not symmetrical, the Duval and Tweedie’s Trim and Fill adjustment was used to estimate the extent of the impact of the bias and what the effect size would have been in the absence of bias. It re-computes the effect size at each iteration until the funnel plot is symmetric about the new effect size. The observed studies are shown as open white circles and the observed point estimate in log units is shown as an open white diamond, while the imputed studies are shown in red open circles and the imputed point estimate in log units is shown as a red diamond. Overall, the impact of bias was probably modest. (TIFF) [file pone.0190823.s002.tiff]
