# Supplementary material for: Liver transplantation for alcoholic hepatitis: A systematic review with meta-analysis
Source: PLoS One. 2018 Jan 11;13(1):e0190823. doi: 10.1371/journal.pone.0190823 (PMC5764315; doi:10.1371/journal.pone.0190823)
Supplement: S1 Table — NA, not applicable. (DOCX) [file pone.0190823.s003.docx]

**S1 Table. Quality assessment of the 11 included studies using the Newcastle Ottawa Scale**

|  | Hanouneh 2014 | Im 2016 | Immordino 2009 | Lee 2017 | Mathurin 2011 | Shakil 1997 | Siddachari 2014 | Singal 2012 | Tomé 2002 | Van Thiel 1995 | Wells 2007 |
| --- | --- | --- | --- | --- | --- | --- | --- | --- | --- | --- | --- |
| **Selection** |  |  |  |  |  |  |  |  |  |  |  |
| 1) Representativeness of the exposed cohort | * | * | * | * | * | * | * | * | * | * | * |
| 2) Selection of the non-exposed cohort | NA | * | * | * | * | NA | * | * | * | * | * |
| 3) Ascertainment of exposure | * | * | * | * | * | * | * | * | * | * | * |
| 4) Demonstration that outcome of interest was not present at start of study | * | * | * | * | * | * | * | * | * | * | * |
| **Comparability** |  |  |  |  |  |  |  |  |  |  |  |
| 1) Comparability of cohorts on the basis of the design or analysis | NA | * | * | * | * | NA | * | * | * | * | * |
| **Outcome** |  |  |  |  |  |  |  |  |  |  |  |
| 1) Assessment of outcome |  | * | * | * | * | * | * |  | * | * | * |
| 2) Was follow-up long enough for outcomes to occur | * | * | * | * | * | * | * | * | * | * | * |
| 3) Adequacy of follow up of cohorts | * | * | * | * | * | * | * | * | * | * | * |
| **Total score** | 5 | 8 | 8 | 8 | 8 | 6 | 8 | 7 | 8 | 8 | 8 |

* A study can be awarded a maximum of one star for each numbered item within the Selection and Outcome categories. A maximum of two stars can be given for Comparability. Points are subsequently summed across eight categories to yield a total score.

Abbreviations: NA, not applicable
