# Supplementary material for: Liver transplantation for alcoholic hepatitis: A systematic review with meta-analysis
Source: PLoS One. 2018 Jan 11;13(1):e0190823. doi: 10.1371/journal.pone.0190823 (PMC5764315; doi:10.1371/journal.pone.0190823)
Supplement: S2 Table — AH, alcoholic hepatitis; CI, confidence interval. (DOCX) [file pone.0190823.s004.docx]

**S2 Table. Results of subgroup analyses including only patients with clinically severe AH or in whom the diagnosis of AH was made on the explants**

|  | Event rate (95% CI) | Heterogeneity |
| --- | --- | --- |
| **Recurrent alcohol consumption after liver transplantation** |  |  |
| **Patients with clinically severe AH** |  |  |
| Pooled estimate rate for recurrent alcohol consumption | 0.20 (0.07-0.43) | p<0.001, *I^2^*=84% |
| Pooled estimate rate for recurrent alcohol consumption in sensitivity analysis excluding studies that did not stringent criteria for selecting candidates for liver transplantation | 0.14 (0.08-0.23) | p=0.6, *I^2^*=0% |
| Pooled estimate rate for recurrent harmful alcohol consumption | 0.15 (0.07-0.27) | p=0.4, *I^2^*=3% |
| **Patients in whom the diagnosis of AH was made on the explants** |  |  |
| Pooled estimate rate for recurrent alcohol consumption | 0.23 (0.11-0.43) | p=0.09, *I^2^*=54% |
| Pooled estimate rate for recurrent alcohol consumption in sensitivity analysis excluding studies that did not use stringent criteria for selecting candidates for liver transplantation | 0.19 (0.08-0.38) | p=0.1, *I^2^*=54% |
| **6-month survival** |  |  |
| **Patients with clinically severe AH** |  |  |
| Pooled estimate rate for 6-month survival | 0.80 (0.69-0.88) | p=0.2, *I^2^*=30% |
| **Patients in whom the diagnosis of AH was made on the explants** |  |  |
| Pooled estimate rate for 6-month survival | 0.90 (0.76-0.97) | p=0.06, *I^2^*=60% |
| Pooled estimate rate for 6-month survival in sensitivity analysis excluding studies that did not use stringent criteria for selecting candidates for liver transplantation | 0.91 (0.72-0.98) | p=0.02, *I^2^*=73% |

Abbreviations: AH, alcoholic hepatitis; CI, confidence interval
