# Supplementary material for: Liver transplantation for alcoholic hepatitis: A systematic review with meta-analysis
Source: PLoS One. 2018 Jan 11;13(1):e0190823. doi: 10.1371/journal.pone.0190823 (PMC5764315; doi:10.1371/journal.pone.0190823)
Supplement: S1 Appendix — (DOCX) [file pone.0190823.s005.docx]

**S1 Appendix. Search strategy used for the identification of eligible studies for the meta-analysis**

Medline (PubMed), Embase, Cochrane library, and manual searches were combined and last performed on June 10^th^, 2017.

**1/ PubMed**

- “liver transplantation” AND “alcoholic hepatitis”: n=135
- “liver transplantation” AND “abstinence”: n= 187
- “liver transplantation” AND “alcohol relapse”: n=56

79 references were identified several times in PubMed. Hence, the search strategy identified 299 references in PubMed.

**2/ Embase**

- “liver transplantation” AND “alcoholic hepatitis”: n=420
- “liver transplantation” AND “abstinence”: n=116
- “liver transplantation” AND “alcohol relapse”: n=431

304 references were identified several times in Embase. Hence, the search strategy identified 763 references in Embase.

**3/ Cochrane library**

- “liver transplantation” AND “alcoholic hepatitis”: n=42
- “liver transplantation” AND “abstinence”: n=6
- “liver transplantation” AND “alcohol relapse”: n=7

12 references were identified several times in Cochrane library. Hence, the search strategy identified 43 references in Cochrane library.

**4/ Manual searches** through references of some review articles: n = 25
